# The forgotten smoker: a qualitative study of attitudes towards smoking, quitting, and tobacco control policies among continuing smokers

**DOI:** 10.1186/1471-2458-13-432

**Published:** 2013-05-03

**Authors:** Navneet Uppal, Lion Shahab, John Britton, Elena Ratschen

**Affiliations:** 1 UK Centre for Tobacco Control Studies, University of Nottingham, Clinical Sciences Building, City Hospital, Nottingham NG5 1PB, UK; 2 Health Behaviour Research Centre, Department of Epidemiology & Public Health, UCL, Gower Street, London WC1E 6BT, UK

**Keywords:** Attitudes, Smoking, Motivation to quit, Tobacco policies, Stop Smoking Services

## Abstract

**Background:**

Although research suggests that the majority of smokers want to quit smoking, the uptake of Stop Smoking Services, designed to assist smokers with quitting, remains low. Little is known about continuing smokers who do not access these services, and opportunities to influence their motivation and encourage quit attempts through the uptake of services. Using PRIME theory, this study explored differences between continuing smokers who had varying levels of motivation to quit, in terms of their plans to quit, evaluative beliefs about smoking, cigarette dependence, and attitudes towards tobacco control policies and services.

**Methods:**

Twenty-two current smokers, recruited from the community, were classified by motivation level to quit using a self-report questionnaire (two groups: high/low). Four focus groups (n=13) and individual interviews (n=9) were conducted with both groups using an interview guide incorporating aspects of PRIME theory. Discussion areas included motives for smoking, attitudes towards smoking and quitting, perceptions of dependence, motives for quitting, barriers to quitting, and attitudes towards existing and impending tobacco control policies and services. Verbatim transcripts were analysed using thematic framework analysis.

**Results:**

All participants expressed low motivation to quit during discussions, despite some initially self-classifying as having high explicit levels of motivation to quit. Both groups reported similar attitudes towards smoking and quitting, including a perceived psychological addiction to smoking, positive evaluations about smoking which inhibited plans to quit, and similar suggested methods to increase motivation (simply wanting to, save money, improve health). Most felt that they ‘ought’ to quit as opposed to ‘wanted’ to. Little influence was ascribed towards tobacco control policies such as plain packaging and hidden sales displays, and participants felt that price increases of tobacco products needed to be considerable in order to influence motivation. Highly motivated smokers expressed more willingness to visit Stop Smoking Services, although none had done so.

**Conclusion:**

Continuing smokers’ attitudes towards smoking and quitting suggests that research and policy need to focus on increasing smokers’ implicit motivation to quit smoking, even for those who classified themselves as having high motivation to quit. Targeted information and further education about Stop Smoking Services is required to increase uptake.

## Background

As in other developed countries, smoking remains the single largest cause of preventable deaths in England, with over 86,000 deaths per year caused by this harmful addictive behaviour [1], and an enormous financial burden of £5bn placed on the NHS [2]. Reducing smoking prevalence and preventing smoking uptake have therefore been identified as important public health targets [3], with increasingly comprehensive measures of tobacco control policy (including smokefree legislation, increased taxation and advertising restrictions [4]) and standards for clinical practice [5] aimed at achieving this goal. Although smoking prevalence has been decreasing steadily over the last decades, the decrease appears to have stagnated in recent years, with 21% of the population currently still smoking [6]. Evidence suggests that the majority of current smokers (70%) are motivated to quit [7]; however, it is only a small minority of smokers (4% each year) who access the NHS Stop Smoking Services (SSS) [8] to assist them, which provide evidence-based behavioural and pharmacological support [9]. Although some research has shown that successful quit attempts may be unplanned [10] or made without any form of pharmacological and/or behavioural support [11,12], other research has demonstrated the benefits of an assisted approach in encouraging quit attempts [13,14]. However, NHS SSS are known to attract smokers with a relatively high level of motivation to quit, as these smokers take the initiative to access services for assistance in quitting [15], whereas the population of smokers who report general motivation to give up, but do not access services, remain underserved in both research and practice; these smokers may be coined ‘forgotten smokers’.

In order to understand the potential of supporting behavioural change in continuing smokers who are not engaged in current quit attempts, the exploration of views and attitudes towards smoking, quitting, and measures of tobacco control is important. Theories of behaviour change espouse the central importance of an individual’s motivation for processes by which an addicted person may quit an addiction. For example, PRIME (*P*lans, *R*esponses, *I*mpulses, *M*otives, *E*valuations) theory [16] of motivation, which has been applied specifically to smoking, states that the decision to quit smoking is based on a smoker’s evaluative beliefs about smoking (either positive or negative), which influence motives to either continue or quit. This motivation then interacts with internal tensions (impulses and urges to smoke) and external triggers (e.g. cues in the environment) to determine subsequent behaviour. Policy measures such as hidden sales displays and smokefree legislation may reduce the environmental cues to smoke, and other measures such as graphic warning imagery may enhance the cues to refrain from smoking. All of this is embedded in a smoker’s overall plan about smoking or quitting (i.e. their overall intentions or rules). While PRIME theory [16] has been applied to smokers in attempting to explain how quit attempts are made, little is known about the spectrum of continuing smokers, and about internal and external factors that may contribute to changes in motivational levels across this spectrum of different ‘types’ of smokers.

Using the conceptual framework of PRIME theory [16], this study aimed to explore aspects related to continuing smokers’ motivation to quit, with a focus on individuals’ overall plans to continue/quit smoking, evaluative beliefs about smoking and quitting, cigarette dependence, attitudes towards various measures of existing and impeding tobacco control (including packaging of cigarettes and hidden sales displays), and attitudes towards NHS SSS. Aiming ultimately at the development of a typology of smokers to match a range of targeted clinical and policy interventions in support of quitting smoking, a qualitative design was adopted to explore smokers’ beliefs in more detail in a smaller community sample, and to identify the key similarities and differences between continuing smokers with varying levels of motivation to quit.

## Methods

### Study design

Qualitative focus groups and semi-structured face-to-face interviews were conducted with continuing smokers.

### Study participants and recruitment

A purposive approach was used to recruit a community sample of smokers aged over 18 who were not currently engaged in quit attempts, but had varying levels of motivation to quit in the future. Participants were recruited from a variety of residential areas across Nottingham and included a range of demographic factors (Aspley, Wollaton, Dunkirk, and Lenton). Recruitment was aimed to continue until theoretical saturation [17] was reached, i.e. no new meaningful data were being obtained. Advertising posters inviting smokers to discuss their opinions about smoking, with wording aiming to recruit both ‘happy’ smokers and those ‘wanting to quit’, were placed in community centres, public houses, libraries, supermarkets, and post offices. A snowballing approach was also used to support recruitment. The study was approved by the ethics board within the Medical School at the University of Nottingham. Participants received £10 as reimbursement for their efforts.

### Study instruments

Participants were initially screened to determine motivation level to quit smoking using the validated Readiness to Quit Ladder [18]; a scale of items 1-10 (‘I have quit smoking’ to ‘I have decided not to quit smoking for my lifetime, I have no interest in quitting’). It was decided a priori by the researchers to classify those with a score of below 6 as having low motivation to quit, and those with a score of 6 and above as having high motivation to quit; previous research has found an average mean value ranging between 5.33 and 5.57 [19]. Participants also completed a short baseline questionnaire designed to gather quantitative data on demographic factors and details of previous quit attempts. Cigarette dependence was assessed using the Fagerstrom Test for Cigarette Dependence (FTCD) [20]. An interview guide was developed to explore a variety of factors to assist in identifying the characteristics and attitudes of both smokers with high and low motivation to quit, and included concepts from PRIME theory [16], such as details of plans to continue/quit smoking and evaluative beliefs about smoking, and several other factors designed to understand why smokers continue to smoke, and methods which may encourage motivation to quit and uptake of NHS SSS (e.g. discussion of the influence of policy measures such as taxation of cigarettes and the impact of this on motivation to quit). Areas for discussion included: motives for smoking, attitudes towards smoking and quitting, motives for quitting, barriers to quitting, and attitudes towards existing and impending policies (e.g. hidden sales displays, plain packaging) and services (e.g. local NHS SSS).

### Procedure

An initial meeting was set up with each participant, prior to arranging a date and time for the focus group, to complete the baseline questionnaire and to screen according to their motivation score [18] (categorised into either low motivation smokers or high motivation smokers). Throughout the course of the focus groups, the researcher felt that group dynamics may have inhibited some responses by participants, as anecdotal evidence from post-focus group discussions with individual participants revealed further details about smoking, which could have been usefully discussed within the focus group itself. This may have been due to a variety of factors related to focus group dynamics and individuals’ confidence in speaking in front of a group of unfamiliar individuals; hence, the study design was adapted to include semi-structured face-to-face interviews with both groups of smokers, to allow for participants to speak freely about their experiences in a more confidential manner. It was felt that both formats, focus groups and individual interviews, were useful to address the research question. Both focus groups and interviews took place at publically accessible locations for participants including community centres. During focus groups and interviews, and throughout data analysis, the researcher critically reflected on her own role in the communicative process, aiming to reduce social desirability effects by creating an environment in which participants felt free to express their own thoughts and beliefs without a sense of judgement.

Focus group lasted for approximately 45 minutes, and interviews for approximately 20-30 minutes, and were recorded using a dictaphone. The researcher followed the interview schedule to cover pre-defined themes for discussion, and allowed for novel themes to emerge freely in both focus groups and interviews.

### Data analysis

Both focus group and interview data were analysed in accordance with thematic Framework Analysis [21,22] to allow for themes to emerge from the data, alongside analysing pre-existing concepts inferred by the researcher (such as differences in motivation level). Separate analysis of focus groups and interviews was considered; however, in view of the identical research question and predefined themes explored, this appeared to yield little more than formal value. Whilst maintaining awareness of this context, data were thus analysed together, using an overarching framework. Analysis involved transcribing each interview *verbatim*, and familiarisation with the data through multiple readings of the transcripts. A priori defined themes and emerging key points were developed into a thematic framework table, where each main point was divided into sub-points which were then coded in the transcripts. Analysis was undertaken manually, and in order to identify within which group of participants each point occurred, the transcript data were then synthesised and charted into the table so that each key point contained details of each participants’ response to it. Interpretative analysis [21] involved grouping together similar key points to identify recurrent themes which revealed attitudes towards smoking and quitting in both groups, and any differences which occurred between the two groups.

## Results

A total of 22 participants (12 classified as low motivation smokers and 10 as high motivation smokers) were recruited until saturation was reached. 13 smokers participated in focus groups (4 groups held in total) and 9 performed individual interviews.

Although not significantly so, cigarette dependence scores were lower in low motivation smokers compared with high motivation smokers. Participants did not differ on other demographic or smoking-related characteristics as a function of their motivation level (Table 1).

**Table 1:** Participant demographics by motivation level

**Variable**	**Low motivation (n=12)**	**High motivation (n=10)**
Male % (N)	66.7 (8)	50 (5)
Mean (SD) Age	31.2 (8.5)	30 (12.6)
Ethnicity % White (N)	91.7 (11)	80.0 (8)
Education % GCSE (N)	58.3 (7)	40.0 (4)
Education % Further (N)	41.67 (5)	60.0 (6)
Mean (SD) Cigarettes per day	11.8 (6.0)	14.9 (8.6)
Mean (SD) Motivation score	4.5 (0.5)	6.7 (1.2)*
Mean (SD) FTCD	2.5 (2.1)	4 (2.8)
% (N) Tried to quit before	66.6 (8)	80 (8)
Mean (SD) Length of last quit attempt (months)	13.2 (16.4)	13.1 (17.5)
Quit method % NRT (N)	37.5 (3)	25.0 (2)
Quit method % Cold turkey (N)	62.5 (5)	75.0 (6)

* t (20) = -5.91, p < .01.

Although low motivation smokers had scored a mean motivation score [18] of 4.5 and high motivation smokers of 6.7 during initial screening, this difference was not reflected during focus groups or interviews. On further exploration by the facilitator, members of both groups were revealed to have low motivation to quit smoking in the immediate context, but had some thoughts about quitting ‘one day’ in the future:

*“Oh, yeah, certainly. One day… it’s easier said than done isn’t it?” (High motivation smoker, Male, Age 57, Interview*).

“*Part of the reason I don*’*t think about quitting that much is that I*’*ve always kind of considered that it will be something that I*’*ll do in the natural course of things*.” (*High motivation smoker*, *Male*, *Age 22*, *Interview*).

“*I*’*m just biding my time*. *Don*’*t know when*, *could be ten years*, *or three years*, *or 15 years*.” (*Low motivation smoker*, *Male*, *Age 40*, *Focus Group*).

As the data revealed no differences between the two predefined groups in terms of their motivation to quit smoking (indicating a potential lack of reliability of the screening instrument in this group of smokers), the three main themes identified through the thematic analysis process - plans to quit smoking; evaluative beliefs about smoking; and perceived effectiveness of tobacco control policies and services on individual smoking behaviour - are presented with illustrative quotes for both groups, with differentiation between groups highlighted only where dictated by findings.

### *P*lans to continue/quit smoking (PRIME theory)

#### Barriers undermining motivation to quit

In accordance with PRIME theory [16], several barriers to quitting were apparent which may have reduced motivation to quit, and enhanced motivation to continue to smoke as part of smokers’ overall plans. The main reasons given as to why smokers did not want to quit immediately were that they felt no detrimental health effects, they enjoyed smoking a lot, and that they would eventually quit in the future. Low motivation smokers also noted that they simply did not want to quit enough to actually do it:

“*Health reasons will be the main reason I give it up*, *but right now I don*’*t even think about it* … *I*’*m quite happy smoking*, *I like smoking*” (*High motivation smoker*, *Female*, *Age 23*, *Focus group*).

“*I*’*ve not had any real reason to I suppose*. *No driving reason to stop doing something I enjoy*” (*Low motivation smoker*, *Male*, *Age 25*, *Interview*).

“*I*’*ve been thinking about it*, *but I don*’*t want to stop*. *That*’*s the point*.” (*Low motivation smoker*, *Male*, *Age 33*, *Focus Group*).

Probing deeper into motivation for quitting, many stated that any desire to quit was based more on what they ‘ought’ to do, rather than what they actually ‘wanted’ to do; as PRIME theory [16] states, ‘wanting’ to change behaviour is a fundamental factor required to elicit behaviour change:

[*Do you want to stop*?] “*Erm* … *I*’*m not sure*. *Maybe*. *I think I should stop*, *but I don*’*t think I will*.” (*Low motivation smoker*, *Female*, *Age 24*, *Interview*).

“*No*, *I don*’*t want to stop*. *But I know I have to stop*.” (*High motivation smoker*, *Female*, *Age 23*, *Focus Group*).

#### Factors likely to increase motivation to quit

The main methods believed by participants of both groups to increase motivation to quit in the future were: ‘wanting’ to quit enough, emergence of detrimental health effects, financial concerns, pregnancy/starting a family, and social disapproval.

“*It*’*s just wanting to do it and having the willpower to do it*. (*High motivation smoker Female*, *Age 24*, *Interview*).

“*I don*’*t want to be one of those people who are in their 60s who have an artificial voice box*, *it*’*s not good*.” (*Low motivation smoker*, *Male*, *Age 22*, *Interview*).

“*It*’*s like burning money away*. *Three and half*, *four and a half grand a year if I smoke 10 to 20 fags a day*.” (*Low motivation smoker*, *Male*, *Age 25*, *Focus Group*).

During discussions, smokers were very quick in identifying these factors, and appeared confident that they would quit ‘one day’ due to any one of the reasons they identified.

### *E*valuative beliefs about smoking

As PRIME theory [16] states, evaluative beliefs about smoking can impact upon a smoker’s motives and desires to continue/quit smoking. As such, the following sub-themes represent smokers’ evaluative beliefs about smoking:

#### Reasons for smoking

The most common reasons for smoking mentioned were enjoyment of smoking, boredom, force of habit, dependency, stress, seeing others smoke, and association with alcohol. In discussions, smokers were very keen to openly discuss why they liked smoking. Additionally, low motivation smokers noted more practical reasons than high motivation smokers for smoking, such as having more breaks at work and something to do with their hands:

“*If you*’*re at work and smoke*, *you tend to get a lot more breaks at work*. *I work in a job where I don*’*t get many breaks anyway*, *apart from smoking*.” (*Low motivation smoker*, *Male*, *Age 25*, *Focus Group*).

All smokers stated they were addicted to smoking in some manner; however, the perceived nature of this addiction differed. Although many noted the biological addiction to nicotine, most thought themselves to be more psychologically addicted to the habit of smoking. During discussions, smokers disagreed about the nature of their addiction, and were willing to discuss this freely with other group members:

“*It might be a psychological addiction where I have a pint in my hand and think* ‘*I*’*ll have a fag*’. *Whereas if I have a bottle of beer at home*, *I won*’*t take a cigarette*.” (*Low motivation smoker*, *Male*, *Age 40*, *Focus Group*).

“*I*’*d always say mine is habitual rather than dependence*. *It*’*s a routine that I do every day*.” (*High motivation smoker*, *Female*, *Age 23*, *Focus Group*).

#### Perceptions of being a smoker

Many smokers, in particular low motivation smokers, had positive evaluations about being a smoker and stated that they enjoyed being a smoker. In some focus groups, smokers encouraged each other to recall stories of smoking experiences, and to further highlight the benefits of being a smoker and things they would miss if they quit. Perceived benefits of being a smoker across both groups were that it was sociable, provided an opportunity to escape, and there was a clear in-group favouritism towards other smokers:

“*I*’*m very suspicious of non*-*smokers*… *you go to a pub and like the non*-*smokers are always a bit square*, *and a bit boring*, *and the smokers are always having a good time*.” (*Low motivation smoker*, *Female*, *Age 35*, *Focus Group*).

“*I*’*d probably miss the social aspect of it*. *There*’*s something about being a smoker in a social situation because you*’*re in that group and there*’*s kind of a nice side to it*.” (*High motivation smoker*, *Male*, *Age 29*, *Interview*).

By contrast, some participants illustrated their dislike of being a smoker, in one case in the context of expressing disapproval of a relapse following a successful quit attempt:

“*I don*’*t like being a smoker myself*, *especially as I*’*d quit*, *and then ended up smoking again*.” (*High motivation smoker*, *Female*, *Age 28*, *Focus Group*).

Smokers stated that if they were to quit, they would miss the physical action of smoking and the social aspect associated with smoking; thus reinforcing their positive evaluations of being a smoker:

“*I just like the action of smoking*, *especially when I*’*m drinking*.” (*Low motivation smoker*, *Male*, *Age 25*, *Interview*).

#### Cognitive dissonance of attitudes towards smoking

Cognitive dissonance, whereby smokers held beliefs about smoking which conflicted with their behavioural actions and led to rationalisation of their behaviour, was apparent in many smokers. Although most smokers enjoyed smoking, some negative attributes of smoking were noted, mainly with high motivation smokers regarding moral norms:

“*I would never smoke around children*. *Never*. *And I don*’*t like smoking around people who don*’*t smoke*.” (*High motivation smoker*, *Female*, *Age 23*, *Focus Group*).

Although some negative attributes were noted, these were not sufficiently compelling to encourage a quit attempt in the immediate future. Furthermore, all smokers stated reasons why they ought to quit smoking, most commonly for their health, and to save money:

“*Logically we should all quit because it*’*s stupid killing yourself*” (*Low motivation smoker*, *Male*, *Age 40*, *Focus Group*).

However, these reasons were often counteracted with statements to justify their smoking habit, and positive appraisals of their behaviour related to positive reasons for smoking; thus reinforcing smokers’ continuation of smoking behaviour and inhibiting the opportunity for making a quit attempt:

“*Even if you don*’*t do anything you*’*re going to die aren*’*t you*? *It*’*s the old joke isn*’*t it*? *If you give up drinking*, *smoking*, *relationships*, *will you live longer*? *No*, *it*’*ll just seem like it*” (*Low motivation smoker*, *Male*, *Age 48*, *Focus Group*).

“*I would feel that I definitely ought not to smoke*, *but that just makes it more attractive*.” (*High motivation smoker*, *Male*, *Age 22*, *Focus Group*).

“*If I took away the element of it being carcinogenic and all the detrimental impacts it has on your life*, *I*’*d say I enjoy it*.” (*High motivation smoker*, *Male*, *Age 22*, *Interview*).

### Perceived effectiveness of policies and services

#### Attitudes towards tobacco control policies

As PRIME theory [16] states, environmental cues can influence the decision to smoke by triggering impulses. However, point of sale displays and packaging of cigarettes were perceived to have little effect on smokers’ purchasing behaviour, as price, taste, and brand familiarity were said to influence purchases the most. Additionally, proposed tobacco control policies were believed to be ineffective in affecting purchases for most smokers. Plain packaging of cigarettes was predicted to have no effect on the brand or quantity of cigarettes bought, neither were hidden sales displays, but these policies were noted to have some potential in deterring younger smokers or preventing impulse purchases:

“*If people think the same way as I think*, *you*’*re going to buy cigarettes whether they*’*re hidden under the counter or there in front of them*.” (*High motivation smoker*, *Female*, *Age 28*, *Focus Group*).

“*It* [*hidden sales displays in Canada*] *really inhibited you from buying something spur of the moment*. *You really needed to know exactly what you wanted*.” (*High motivation smoker*, *Male*, *Age 22*, *Interview*).

“*It will work from the point of view of some kids who are drawn to shiny things* … *but I don*’*t think it*’*s going to make much difference to established smokers*.” (*Low motivation smoker*, *Male*, *Age 40*, *Focus Group*).

Many smokers stated that price increases of cigarettes needed to be more drastic in order to effectively reduce the number of purchases made:

“*The incremental rises are pathetic*, *it doesn*’*t deter anyone*”. (*Low motivation smoker*, *Male*, *Age 40*, *Focus Group*).

“*If it gets to* £*4 a pint*, *I won*’*t drink alcohol*. *And then it gets to four and you*’*re like* ‘*if it gets to* £*5*, *that will be the final straw*’. *And it*’*s the same with cigarettes* … *you find a way*”. (*Low motivation smoker*, *Male*, *Age 48*, *Focus Group*).

[*Would you keep paying it*?] “*Yeah*, *I think I probably would*. *Because the inclinations are so small really*. *It*’*s not going to go from* £*4 to* £*10*, *it*’*s slow*, *incremental*.” (*High motivation smoker*, *Female*, *Age 23*, *Focus Group*).

#### Attitudes towards NHS SSS

Differences between groups were apparent in attitudes towards NHS SSS. Low motivation smokers were more dismissive towards these stating that quitting was ‘a personal thing’, and were less willing to use such services, whereas high motivation smokers appeared generally more appreciative of the assistance on offer, and although none had actually used NHS SSS, they stated they would use it if they felt the need to (e.g. to combat the nicotine addiction using harm reduction methods or if an unassisted quit attempt had failed). However, during discussions, it was apparent that there was a clear lack of knowledge regarding what NHS SSS were, and how to access them. Only after the facilitator had explained what they offered, was there some appreciation and willingness to use a service if required:

“*I think if I seriously wanted to quit*, *and I thought that I wouldn*’*t be able to do it myself*, *I would be very willing to go and use that kind of service*.” (*High motivation smoker*, *Female*, *Age 23*, *Interview*).

“*If I was going to try and stop the nicotine addiction*, *then it would be a good idea to go to somebody who knows what they*’*re doing*.” (*High motivation smoker*, *Male*, *Age 29*, *Interview*).

When asked about the potential quit methods smokers would most likely use, the majority across groups stated that they would prefer to ‘go cold turkey’:

“*I*’*d be quite happy going cold turkey and seeing what happens*.” (*Low motivation smoker*, *Male*, *Age 25*, *Interview*).

“*I think I*’*d probably wake up and think* ‘*right*, *last packet of cigarettes*’ *and then no more*.” (*High motivation smoker*, *Female*, *Age 24*, *Interview*).

However, some high motivation smokers also suggested being offered a sympathetic and supportive approach by health professionals would help them to quit, suggesting that these smokers might benefit the most from increased encouragement to access NHS SSS:

“*You can go to your doctor or your chemist and there*’*s a lot more encouragement and advice to help you pack in*. *That wasn*’*t true say five years ago*… *That*’*s a far more sympathetic approach than trying to put the cost up*.” (*High motivation smoker*, *Male*, *Age 52*, *Interview*).

#### Attitudes towards NRT

Smokers displayed differing opinions regarding NRT; some believed they were an effective cessation tool whereas others did not:

“*I whacked one on in the morning and was like* ‘*yeah*, *shall we just have a fag*?’ *So we took them off and had a fag*” (*Low motivation smoker*, *Female*, *Age 35*, *Focus Group*).

“*You honestly do not feel the need*, *that craving*, *to smoke*. *It*’*s really difficult to want to smoke while having that patch*.” (*High motivation smoker*, *Male*, *Age 22*, *Interview*).

Many smokers had negative views towards NRT stating that they were concerned about the side effects (including taste and irritability), and also that it simply didn’t work as it was believed to only treat the nicotine addiction and not the habitual aspects of smoking, whereas others liked the relief and confidence it provided; highlighting the individual preferences for quit support that need to be considered by health professionals:

“*The plastic thing that makes you feel sick*. *The sweets are disgusting*.” (*High motivation smoker*, *Female*, *Age 24*, *Interview*).

“*I*’*d probably use other means*, *because it just seems a bit clinical*. *Because I don*’*t smoke for the nicotine*, *I smoke for everything else with it*.” (*High motivation smoker*, *Male*, *Age 22*, *Focus Group*).

“*It does give you the idea that stopping smoking is possible*, *from a position where you think it*’*s going to be really hard*.” (*High motivation smoker*, *Male*, *Age 29*, *Interview*).

## Discussion

In this study, attitudes towards smoking and quitting were similar for both low motivation and high motivation smokers, despite participants demonstrating explicit differences in motivational scores to quit smoking during the screening process. This suggests that more sensitive measures of current levels of motivation to quit need to be developed in further research. The discrepancy between high motivation smokers’ questionnaire-based and interview-based motivation levels may be explained theoretically by explicit and implicit motivations, as research has shown that explicit and implicit attitudes are distinct concepts [23] that may not necessarily be related [24]. Hence, high motivation smokers may appear to be motivated to quit, but their implicit attitudes revealed a liking for smoking and a lower motivation to quit. Furthermore, this may suggest that the estimated figure of 70% of smokers who report motivation to quit [7] may be misleading, as many smokers may lack the implicit motivation needed to quit, which may explain the low uptake of NHS SSS [7].

With regards to the first main theme ‘*P*lans to continue/quit smoking’, according to PRIME theory [16], a strong motive (‘want’), as opposed to a rational thought (‘ought’) to quit is required to inhibit internal impulses to smoke. Smokers are thought to be in a state of motivational tension and if only a rational thought, but no strong motive to quit is present, the impulse to smoke triumphs. Previous research has also found that smokers feel they ‘ought’ to quit, rather than ‘want’ to quit [25]. Generally, smokers’ overall plans were to continue to smoke, with only some thoughts given to quitting in the future. This finding may have practical implications for treatment, as it may be effective for primary care providers to offer brief cessation advice to all patients who smoke during consultations [26]. In this manner, moving away from the traditional Transtheoretical model [27] of only providing support to smokers who are motivated to quit, it may be possible to trigger a quit attempt and referral to NHS SSS through changing a smoker’s overall *P*lan about smoking during a period of motivational tension [28].

In terms of the second main theme of ‘*E*valuative beliefs about smoking’ which can influence smokers’ motives and overall plans, smokers knew the health risks of smoking yet some continued to justify their smoking behaviour through positive appraisals. Research has shown that cognitive dissonance is common in smokers, whereby smokers know the health risks of smoking but rationalise their smoking behaviour to accommodate this [29]. Further research [30] has also shown that self-exempting beliefs are constructed by smokers who do not intend to quit, in order to justify their smoking behaviour (for example, smoking is ‘worth it’). However, in the present study, these beliefs were found in both low motivation and high motivation smokers, suggesting that even smokers who explicitly state having higher levels of motivation to quit, may implicitly hold some self-exempting beliefs which obstruct plans to quit.

Within the third main theme of ‘perceived effectiveness of policies and services’, consistent with other qualitative research [31], participants had unfavourable attitudes towards tobacco control measures and highlighted the perceived ineffectiveness of policies such as plain packaging of cigarettes and hidden sales displays. However, high motivation smokers did note that these policies may deter younger smokers and inhibit impulse purchases. This is consistent with research that has found that only high motivation smokers view plain packaging as an effective strategy to support cessation [32] and that point of sale displays do influence purchases in younger smokers [33] and encourage more smoking in established smokers [34]. Participants noted that any tobacco product price increases need to be substantial in order to have a real impact on motivations, despite research demonstrating the effectiveness of this policy as it currently stands [35]. This finding is consistent with other qualitative research [31], although some research has shown that price increases may lead to an increase in contraband tobacco use in smokers who feel highly addicted [36].

None of the participants had used NHS SSS, but high motivation smokers were more willing to do so, whereas low motivation smokers were more dismissive of this support. Consistent with this study, other qualitative research has also found that smokers have little knowledge of NHS SSS and perceive them to be ineffective [37]. Attitudes towards NRT itself varied greatly with different perceptions of the effectiveness of this cessation tool, with some negative views stating the side effects of NRT and that it is too clinical a treatment. Previous qualitative research has also shown that smokers are wary of the side effects of NRT and have misconceptions regarding the cost of such treatments [31,38]; however, cost was not found to be an inhibiting factor in this study. Although many smokers stated that their preferred quit method was ‘cold turkey’, which, as previously noted [12], may be a successful quit method for some smokers, other research has suggested that further education to increase knowledge of the effectiveness and accessibility of available cessation tools may be beneficial to smokers who need support [38] and thus, might potentially increase the uptake of NHS SSS and assist more smokers in quitting.

Further research of the association between the characteristics of continuing smokers who have not accessed NHS SSS, and their susceptibility towards different interventions and policy measures by quantitative means is required to assist in the development of a typology to target measures to increase motivation to quit across the spectrum of continuing smokers, and to support the further decrease of smoking prevalence rates in the future.

### Study limitations

There were high levels of non-attendance at some focus groups, and inhibited responses by some participants within focus groups which may have restricted discussions; however, individual interviews were conducted to compensate for the loss in recruitment and to provide an opportunity for more in-depth open discussion with individuals who may otherwise have felt restricted speaking in a group. A small sample of smokers were recruited which limits generalizability; however, this was the first exploratory step towards informing the development of a typology of smokers, and it is recommended to recruit a larger sample and conduct a quantitative study in order to replicate and further generalise these results.

## Conclusion

This study found that despite some smokers self-classifying as having high motivation to quit, during discussions, they were revealed to have low motivation to quit in the immediate future. As such, the discrepancy between explicit and implicit motivations needs to be further researched. Smokers felt they ‘ought’ to quit smoking rather than ‘wanted’ to, thus highlighting the need to identify personally relevant levers to increase motivation to quit. Smokers felt that price increases of tobacco products needed to be more drastic in order to influence motivation and reduce the number of purchases. Increased knowledge of NHS SSS is required to increase uptake in smokers who may require support, but are unaware of the pharmacological and behavioural support that is available within these services to combat both the physical and psychological aspects of smoking.

## Competing interests

NU, JB, and ER have no competing interests. LS has received an honorarium for a talk and travel expenses from a pharmaceutical company making smoking cessation products.

## Author’s contributions

NU conducted the interviews, analysis, and drafted the manuscript under supervision from ER, LS, and JB who supported the interview guide development, reviewed the analysis and results, and revised the manuscript draft. All authors read and approved the final manuscript.

## Pre-publication history

The pre-publication history for this paper can be accessed here:

http://www.biomedcentral.com/1471-2458/13/432/prepub
